# Incidence, clinical spectrum, and immunotherapy of non-ischemic cerebral enhancing lesions after endovascular therapy

**DOI:** 10.1177/17562864211072372

**Published:** 2022-01-31

**Authors:** Antonios Bayas, Monika Christ, Ansgar Berlis, Markus Naumann, Michael Ertl, Felix Joachimski, Mona Müller, Julia Welzel, Lisa Ann Gerdes, Klaus Seelos, Christoph Maurer

**Affiliations:** Department of Neurology, University Hospital of Augsburg, Stenglinstraße 2, D-86156 Augsburg, Germany; Department of Neurology, University Hospital of Augsburg, Augsburg, Germany; Department of Neuroradiology, University Hospital of Augsburg, Augsburg, Germany; Department of Neurology, University Hospital of Augsburg, Augsburg, Germany; Department of Neurology, University Hospital of Augsburg, Augsburg, Germany; Department of Neuroradiology, University Hospital of Augsburg, Augsburg, Germany; Department of Neurology, University Hospital of Augsburg, Augsburg, Germany; Department of Dermatology, University Hospital of Augsburg, Augsburg, Germany; Institute of Clinical Neuroimmunology, Biomedical Center and University Hospital, LMU Munich, Munich, Germany; Institute of Neuroradiology, University Hospital, LMU Munich, Munich, Germany; Department of Neuroradiology, University Hospital of Augsburg, Augsburg, Germany

**Keywords:** aneurysm, endovascular therapy, MRI, NICE, non-ischemic cerebral enhancing lesions

## Abstract

**Background:**

Symptomatic and asymptomatic delayed non-ischemic cerebral enhancing (NICE) lesions in magnetic resonance imaging (MRI) have been reported as a rare complication after endovascular therapy (EVT) in recent years with incidence rates between 0.05% and 0.9% in most studies. Information on long-term clinical course and immunotherapies is scarce or has not been reported in detail in the literature. Objective: Aims of our study were to assess the incidence of NICE lesions in patients after cerebral EVT over a period of more than 12 years, describe clinical and EVT characteristics, and immunotherapies applied.

**Methods:**

A retrospective chart review of all patients treated by endovascular therapy for symptomatic or asymptomatic aneurysms at the University Hospital of Augsburg from May 1, 2008 to December 31, 2020 was performed. Patients were identified retrospectively and followed-up prospectively where appropriate. In addition, one case treated at another institution was included.

**Results:**

Five out of 746 patients, 0.67%, developed NICE lesions after EVT, all with non-ruptured aneurysms and all symptomatic upon detection of NICE lesions by MRI. In total, the disease course of 6 female patients is reported. Symptoms occurred after a mean time of 15 days (±13.42, SD) after EVT with headache (6/6 patients), focal neurological signs (6/6 patients), epileptic seizures (2/6 patients) and cognitive deficits (3/6 patients). All 6 patients received glucocorticosteroids (GCS), 1/6 azathioprine (AZA), 4/6 mycophenolate mofetil (MMF), 1/6 methotrexate (MTX), 1/6 rituximab (RTX), 2/6 cyclophosphamide (CYC) and 3/6 tocilizumab (TCZ). A treatment response could be observed for GCS, TCZ and MMF (in two of four cases), RTX and AZA did not result in disease stabilization.

**Conclusions:**

Delayed NICE lesions are a rare complication after EVT, requiring immunotherapies in all patients reported here. Physicians should be aware of this disorder in case of new symptoms or contrast enhancing lesions after EVT.

## Introduction

Standard of care for treatment of cerebral aneurysms has been dominated by endovascular therapy (EVT) in recent years. Main complications of endovascular coiling are procedural aneurysm perforations and thromboembolic events.^1^ Delayed non-ischemic cerebral enhancing (NICE) lesions in magnetic resonance imaging (MRI) have rarely been reported as a complication after EVT.^2–8^ Punctate, nodular or annular foci of leptomeningeal, cortical, and subcortical enhancements and perilesional edema^8^ in the territory of the endovascular access of EVT represent characteristics of NICE lesions. The disorder has also been termed as delayed cerebral hypersensitivity,^4^ leukoencephalopathy^9^ or descriptively as reversible intracranial parenchymal changes.^7^ Incidence rates after EVT are reported to be as high as 0.05%^10^ to 0.9%,^11^ except for one study reporting a higher incidence rate of 2.3%.^12^ Several studies have been published on the pathogenesis of NICE lesions: Besides foreign body emboli, in some patients identified as hydrophilic polymer coating emboli, and subsequent granulomatous reactions,^2,3,5,6,8,13,14^ cerebral metallic hypersensitivity and nickel allergy^4,7,15^ have been reported. Successful treatment with glucocorticosteroids (GCS) has been described in many cases, other immunosuppressants like mycophenolate mofetil (MMF) or azathioprine (AZA) in few cases only.^13,16,17^ However, data on long-term follow-up and response to long-term treatments are scarce and have not been reported in detail in many cases.

Main aims of our study were (a) to compute the incidence of NICE lesions in patients undergoing cerebral EVT over 12 years and 8 months, (b) describe clinical and EVT characteristics of patients affected, and (c) immunotherapies used, including long-term follow-up.

## Methods

### Patients

#### Incidence of NICE lesions after EVT

A retrospective chart review of all patients treated by endovascular therapy (EVT) for cerebral aneurysms at the University Hospital of Augsburg from 1 May 2008, when standard MRI follow-ups after EVT at our institution were established, to 31 December 2020 was performed. According to the standard follow-up protocol at our institution, all patients are scheduled for a cerebral MRI follow-up after 6 months after EVT; in case of new or worsening clinical symptoms, MRI is performed immediately. As this was a mainly retrospective study, the differential diagnostic workup did not follow a standardized algorithm, but was performed individually at the treating physicians` discretion. All patients with NICE lesions on MRI, either asymptomatic detected by routine MRI follow-up or clinically symptomatic, were included in our analysis.

#### Follow-up of patients with NICE lesions

Patients diagnosed with NICE lesions at the University Hospital Augsburg were identified retrospectively (cases 1–5) and followed-up prospectively (cases 1, 4 and 5) until data cut (for clinical course of cases 1–5) end of September 2021. Furthermore, one patient (case 6; included after personal communication, L.A.G.), diagnosed and treated at the Institute of Clinical Neuroimmunology, Biomedical Center and University Hospitals, Ludwig-Maximilians-University Munich, Germany (LMU Munich), reported in part before,^2^ has been included.

The following data were analyzed: age, gender, history of allergies, location and size of the treated aneurysm(s), material used for EVT, intervention duration, symptoms and their time of onset after EVT, MRI data, clinical course, treatment and, if available, laboratory findings including cerebrospinal fluid (CSF), results of dermatological tests, cognitive tests, electroencephalography (EEG), duplex sonography and digital subtraction angiography (DSA) at onset and during follow-up. None of the patients underwent biopsy or surgical resection of the lesions.

### Statistical analysis

Descriptive statistics were used for the incidence of NICE lesions at the University Hospital of Augsburg for the period 1 May, 2008 until 31 December, 2020, calculated as the number of patients who developed (one or several times) NICE lesions after EVT divided by all patients that were treated with EVT and underwent at least one MRI within the following year. The case treated at the LMU Munich was not included in the computation of incidence.

### Ethics statement

The study at the University Hospital Augsburg was approved by the ethical review committee of the Friedrich-Alexander University Erlangen-Nuremberg (No.: 101_21 B), all patients gave written informed consent. The patient treated at the LMU Munich gave local consent there.

### EVT and MRI

Initial and follow-up images were retrospectively reviewed by two senior neuroradiologists. All endovascular procedures included a standardized angiography of all brain-supplying vessels with assessment of both vertebral arteries and the internal carotid artery in four planes each as well as an additional 3D rotational angiography of the aneurysm-hosting vessel. Treatment decisions and material selection were based on the angiographic results and included a variety of devices including several types of coils, stent- or balloon-assisted coiling, the use of intra- and extra-aneurysmal flow diverters and a wide range of available neck-bridging devices. Standard MR-protocol for patients after endovascular aneurysm therapy included diffusion-weighted imaging (DWI), thin-slice T2-wheighted imaging over the treated aneurysm in axial and coronal planes, MR-angiography with and without intravenous contrast administration and T1-wheighted images of the whole brain.

## Results

### Incidence of NICE lesions after EVT

Within 12 years and 8 months (1 May 2008 to 31 December 2020), a total of 1101 patients treated for cerebral aneurysms by EVT at the University Hospital Augsburg were identified, 513 patients with non-ruptured / incidental aneurysms and 588 patients with ruptured aneurysms. In all, 132 patients received repeated EVTs. Of 1101 patients, 746 patients received at least one follow-up MRI within 1 year after EVT. The mean time to first follow-up MRI, 159 (± 175.2, SD) days after EVT, was shorter than planned by standard follow-up protocol (6 months after EVT), because some patients received MRI earlier due to peri-interventional complications/neurological signs and symptoms such as ischemia, vasospasm, impaired consciousness, headache, symptomatic NICE lesions, and so on. Excluding MRI scans within the first 30 days after EVT performed for reasons other than symptoms related to NICE lesions, 724 patients received at least one follow-up MRI within 1 year after EVT after a mean time of 171 (±71, SD) days after EVT.

**Figure 1.**
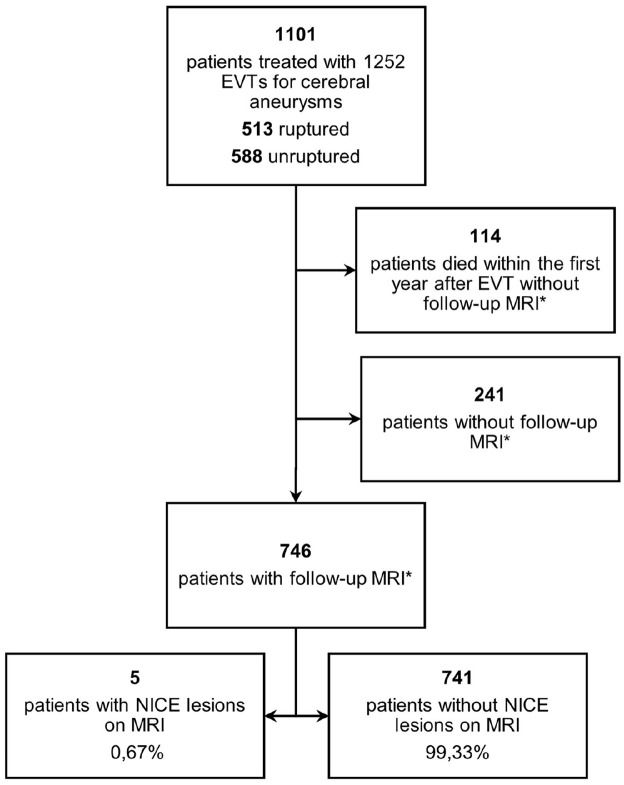
Flowchart of patients included in the study at the University Hospital of Augsburg. EVT, endovascular therapy; MRI, magnetic resonance imaging; NICE, non-ischemic cerebral enhancing. *Follow-up cerebral MRI within the first year after EVT.

At data cut off for incidence computation on August 19, 2021, routine MRI follow-up was pending in 8 patients who had not yet completed the 1-year follow-up period. Five out of 746 patients, 0.67%, developed NICE lesions after EVT, all with non-ruptured aneurysms and all symptomatic upon detection of NICE lesions by MRI. Results are displayed in Figure 1. Taking all patients undergoing EVT, regardless of follow-up MRI, into consideration, 5 out of 1101 patients were affected, resulting in an incidence of 0.45%.

### Disease course of patients with NICE lesions

**Figure 2.**
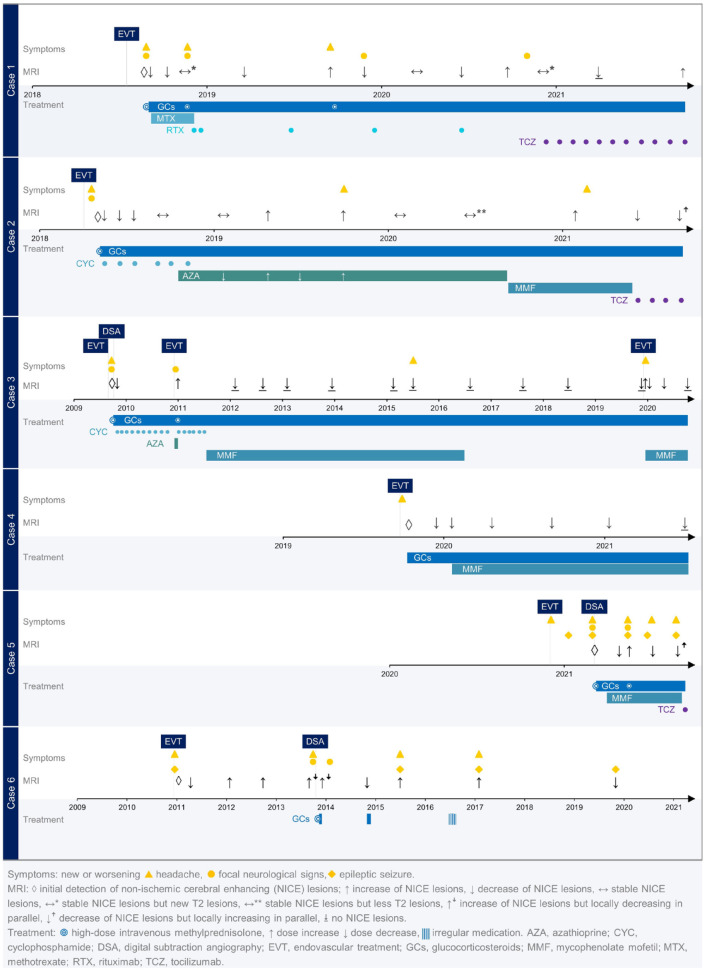
Clinical course of patients.

Figure 2 summarizes the clinical course, MRI activity and treatment of the 6 cases reported. Table 1 shows patient characteristics, as well as MRI and CSF results at time of diagnosis. We report 6 females with a mean age of 51 (±12.12, SD) years. Symptoms occurred after a mean time of 15 days (±13.42, SD; range 2-40 days) after EVT with headache (6/6 patients), focal neurological signs (6/6 patients), epileptic seizures (2/6 patients) and cognitive deficits (3/6 patients) (Table 1), leading to a mild to moderate disability in all patients (4/6 patients modified Rankin Scale (mRS) score 2 and 2/6 patients mRS 3). CSF analysis, where available, revealed a pleocytosis in 2 out of 5 patients (37 and 56 leucocytes/ µl respectively, details see Supplementary Appendix 1). Vascular irregularities suggestive of vasculitis were detected in 3 of the 3 patients who underwent DSA after symptom onset. After diagnosis of symptomatic NICE lesions, all patients received GCS (6/6 patients), 1/6 patients AZA, 4/6 patients MMF, 1/6 patients methotrexate (MTX), 1/6 patients rituximab (RTX), 2/6 patients cyclophosphamide (CYC) and 3/6 patients tocilizumab (TCZ). During a follow-up period of 60.17 ± 48.74 months (mean ± SD; range 9 - 132 months), new symptoms and / or clinical signs and new NICE lesions were documented in 5 out of the 6 patients. Detailed case descriptions of all 6 patients are summarized in Supplementary Appendix 1.

**Table 1.** Patient characteristics, neurological and MRI findings at time of diagnosis.

Case No.	Age, y	Gender, F/M	Symptoms					MRI at onset				CSF
			onset. days after EVT	Headache	Epileptic seizure	Cognitive deficits	Neurological signs	Days after EVT	NICE lesion location	Number of NICE lesions	Lesions outside the vascular territory of the treated aneurysm (if yes. territory angiographed during EVT)?	White cells /µl
1	33	F	40	Yes	No	Yes	Right-sided hemiparesis, aphasia, dysarthria and hypesthesia right hand	41	anterior circulation both sides and potentially^a^ posterior circulation	> 20	Yes (potentially^a^)	2
2	52	F	16	Yes	No	No	Left arm paresis	33	anterior circulation both sides	> 20	Yes (Yes)	37
3	51	F	23	Yes	No	No	Left-sided hemiparesis, left homonymous hemianopsia, neglect	28	anterior circulation both sides	> 20	Yes (Yes)	56
4	73	F	4	Yes	No	Yes	Visual disturbance, dizziness	16	anterior circulation both sides and posterior circulation	> 20	Yes (Yes)	NA
5	43	F	2	Yes	Yes	Yes^b^	Hypesthesia, left hand paresis	96	anterior circulation both sides	> 20	No	3
6	54	F	5	Yes	Yes	No	Recurrent left brachiofacial paresthesias, binocular visual disturbances	5	anterior circulation right side	> 20	No	4

CSF, cerebrospinal fluid; EVT, endovascular treatment; MRI, magnetic resonance imaging; NA, not available; NICE, non-ischemic cerebral enhancing.

a Because of intermediate-type (fetal/adult) posterior cerebral arteries (PCA) both sides, NICE lesions in the PCA territory cannot be reliably assigned to the anterior or posterior circulation.

b Not complained about by the patient, but detected by cognitive testing.

At last follow-up, 5/6 patients still received GCS at a mean prednisolone dose of 7.3 (± 6.49, SD) mg/day, all 5 patients received additional immunotherapies, 3/6 TCZ (8 mg/kilogram body weight every 4 weeks) and 2/6 MMF at a dose of 2 g/day. The patient not receiving any treatment (case 6) at last follow-up, declined GCS or other immunotherapies despite clinical and MRI activity over the course of time. Taken our data together, GCS, TCZ (in 2 of 3 cases, case 5 treated only once so far) and MMF (in 2 of 4 cases) resulted in MRI and / or clinical improvement (see Supplementary Appendix 1 and Figure 2), RTX and AZA did not result in disease stabilization. At last follow-up, all patients had no or only minor disability (mRS 0-1).

### EVT characteristics

All aneurysms were located at typical sites in the Circle of Willis or the bifurcation of the middle cerebral artery. The aneurysms were small or medium sized with the largest diameter less than 12 mm. Inflammatory aneurysms were not observed. A 6Fr Envoy was used as the guiding catheter in all cases, while a wide range of materials were used for microwire, microcatheter and embolization. Of note, no patient underwent coiling alone; in all cases, stent-assisted coiling or flow-diverter treatment was performed. In no case did a vascular occlusion or altered perfusion occur during the course of the procedure. Table 2 summarizes aneurysm characteristics, materials used for EVT and intervention duration. Case 3 was treated three times.

**Table 2.** EVT characteristics.

Case No.		Aneurysm location	Aneurysm size, mm	Symptomatic	Procedure	Guiding catheter	Micro catheter	Micro wire	Stent	Flow diverter	WEB	Coil	Balloon catheter	Closure device	Residual aneurysm	Duration EVT / fluoroscopy time, minutes
1		Left ICA	3.6 x 3.7	No	SACE	Envoy 6 F MPD 90 cm	Excelsior SL 10 Straight	Transend EX 014	Neuroform Atlas 4.0 x 15, Neuroform Atlas 4.5 x 21	None	None	Bare platinum	None	Angio-Seal 8F	Small dog ear	85 / 43
		Right ICA	5.2 x 8.2	No	SACE										Small dog ear	
2		Right MCA	5.5 x 3.7	No	WEB	Envoy 6 F MPD 90 cm	Via 17, Excelsior SL 10 Straight	Synchro 200 cm	LVIS jr. 2.5 x 16	None	WEB 17 SL W5-5-2, WEB 17 SL W5-4-3	Hydrogel- coated	None	Exoseal 7F	No	110 / 55
		Left MCA	3.3 x 3.6	No	WEB										No	
		AcomA	2.5 x 1.8	No	SACE										No	
3	1^st^ EVT	Right MCA	11.7 x 9.0	Yes, MCA stroke	SACE	Envoy 6 F MPD 90 cm	Excelsior SL 10 Straight, Echelon 90°	Transend EX 014, Synchro 300 cm, Synchro 200 cm	Neuroform3 3 x 20	None	None	Bare platinum, hydrogel-coated	None	Angio-Seal 8F	Yes (small)	95 / NA
		Right MCA	1.5 x 1.4	No	SACE										No	
		Right PcaA	5.4 x 3.7	No	SACE										Yes (small)	
	2^nd^ EVT	Left MCA	5.6 x 5.9	No	SACE	Envoy 6 F MPD 90 cm	Excelsior SL 10 Straight Echelon 90°	Transend EX 014, Synchro 200 cm	Neuroform3 4 x 30	None	None	Bare platinum, hydrogel-coated	None	Angio-Seal 8F	No	160 / NA
		Left MCA	4.6 x 4.1	No	SACE										No	
		Left PcaA	1.5 x 1.0	No	not successful										Yes	
		Right MCA (recurrence)	8.8 x 7.8	No	not successful										Yes	
	3^rd^ EVT	Right MCA (recurrence)	10.0 x 8.0	No	Flow diverter	Envoy 6 F MPD 90 cm	Excelsior XT 27	Transend EX 014	None	Surpass Evolve 2.5 x 15	None	None	Scepter C 4 x 15	Angio-Seal 8F	Yes	55 / 25
4		Basilar	9.4 x 7.9	Yes, local space-occupying effect	SACE	Envoy 6 F MPD 90 cm	Excelsior SL 10 Straight Headway 17	Transend EX 014	LVIS EVO 4.0 x 21	None	None	Hydrogel- coated	None	Angio-Seal 8F	Yes (small)	80 / 40
5		Right ICA	7.5 x 9,4	No	SACE	Envoy 6 F MPD 90 cm	Excelsior SL 10 Straight, Headway 17	Transend EX 014	LVIS EVO 4.0 x 27	Fred 4.0 x 23 x 17	None	Hydrogel- coated	None	Exoseal 7F	Low contrast medium inflow	85 / 33
		Left ICA	5.2 x 4.8	No	Flow diverter										no	
6		Right ICA	6.8 x 8.8	No	Flow diverter	Envoy 6 F MPD 90 cm, Fargomax 6 F 115/8	Marksman 150cm	Traxcess 14	None	Pipline 4 x 20	None	None	None	Angio-Seal 6F	Yes	60 / 35

AcomA, anterior communicating artery; EVT, endovascular treatment; F, female; ICA, internal carotid artery; M, male; MCA, middle cerebral artery; MPD, mulitipurpose D; NA, not available; PcaA, pericallosal artery; SACE, stent-assisted coil embolization; WEB, woven endobridge; y, year.

For all patients guide wire Terumo 0.035’’ and sheath Terumo 6 F or 7 F.

For follow-up, a diagnostic angiography was performed only in cases 3, 5 and 6, in cases 3 and 5 showing subtle irregularities of the peripheral arteries in the treated vascular territory leading initially to the diagnosis of vasculitis. In case 6, DSA revealed multiple stenoses, typical for vasculitis, of the distal pial arteries and cortical branches of the right anterior and middle cerebral artery.

### MRI findings

**Figure 3.**
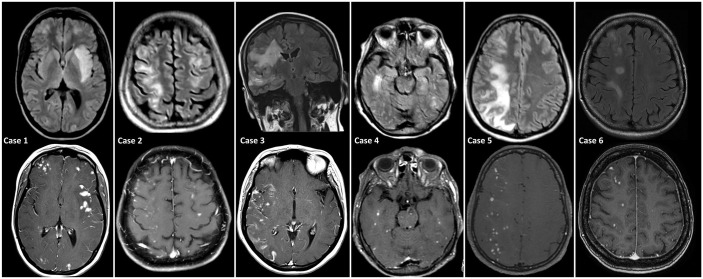
MRI of cases 1 to 5 at initial presentation and follow-up MRI of case 6. The top row shows the T2 FLAIR weighted sequence in each case with partially extensive edema around the lesions. The bottom row shows the typical aspect of NICE lesions after contrast administration with punctate to patchy enhancement in the cortical and subcortical white matter. Case 3 additionally shows cortical gyriform enhancement due to a contemporaneous ischemic stroke.

MRI findings included typically punctuate or patchy contrast enhancement. Linear to cortical enhancement was additionally found in one case as well (case 3), most probably related to overlapping changes of the aneurysm related infarction of a partial thrombosed symptomatic aneurysm. Main focus of the enhancement was the territory of the treated aneurysm/aneurysms. However, in all but one patient, NICE lesions were also detected outside the territory of the treated aneurysm. Albeit, all vascular territories exhibiting NICE lesions were also subjected to DSA. NICE lesions were detected by MRI after 36.5 (±29.03, SD) days. On initial presentation, the lesions demonstrated extensive perifocal edema on T2 weighted images, without clear mass effect and T2-shine through on DWI. No associated hemorrhage was observed. Long-term MRI follow-up is depicted in Figure 2. Figure 3 shows the typical aspect of NICE lesions on T2 Flair and T1-wheighted images after application of contrast media in each case.

## Discussion

EVT of cerebral aneurysms may be complicated by delayed-onset NICE lesions, detected by MRI in rare cases.^2–8^ Here, we report clinical and EVT characteristics, immunotherapies, and follow-up of 6 females who became symptomatic after treatment of asymptomatic cerebral aneurysms exhibiting NICE lesions in MRI.

The disorder has also been described as delayed cerebral hypersensitivity,^4^ leukoencephalopathy^9^ or descriptively as reversible intracranial parenchymal changes.^7^ For ease, in this study MRI lesions described in the literature for this disorder are uniformly named as NICE lesions, though knowing that not all MRI lesions occuring in this disorder are contrast enhancing.

Incidence rates of NICE lesions after EVT have been reported to be as high as 0.05% (31/ 58.815 patients during a period of 13 years),^10^ 0.5% (2/374 patients, treated during a 3-year period)^8^ and 0.6% (5/approximately 720 patients during a 2-year period),^5^ with the highest reported incidence of 2.3% (7/305 patients, treated during a 3-year period).^12^ However, the accuracy of incidence rates in some studies may be limited, because asymptomatic EVT treated patients were not routinely subjected to follow-up MRI or incidence assessment has not been described in detail.^5,8,12^ By using regular follow-up MRIs (after 3, 6, and 12 months), Ikemura *et al.*^11^ found an incidence of delayed leukoencephalopathy of 0.9% (16/1722 patients during a 12-years period), 9 patients were asymptomatic. Asymptomatic lesions were also found in another case series^2^ and in the French national registry^10^ published recently. In our study, 5 out of 746 patients, resulting in an incidence of 0.67%, developed NICE lesions after EVT, all with non-ruptured aneurysms and all symptomatic upon detection of NICE lesions by MRI. This is lower than reported by Ikemura *et al.*,^11^ but in contrast to their cohort, we did not identify asymptomatic patients with NICE lesions in follow-up MRIs. The higher incidence of NICE lesions in the study by Ikemura *et al.*^11^ compared to our data may also be related to a lower number of ruptured aneurysms in their cohort (193 ruptured, 1401 unruptured vs 513 unruptured and 588 ruptured aneurysms in our study) with less fatal outcomes lost to follow-up. In another study, Moreno Estébanez *et al.*^18^ identified 11 symptomatic intracranial embolic foreign-body reactions from a total of 7446 neurointerventional procedures between 2013 and 2019 with an incidence rate of 0.14%. The cumulative incidence raised to 0.45%, if only therapeutic procedures were considered and to 1.02% if only therapeutic procedures with placement of coils or stents were taken into account, with the highest incidence of 1.66% related to aneurysm embolization. These data indicate a higher risk in aneurysm embolization versus thrombectomy. Taken together, our data and those of others groups indicate that NICE lesions represent a quite uncommon complication of EVT, but frequent enough to raise clinicians’ awareness, especially in cases of clinical worsening after EVT.

The mean time from EVT to symptom onset attributable to NICE lesions in our study was 15 days (range 2-40 days). In previously published cases, a mean delay to symptoms’ onset or discovery of asymptomatic lesions of 12 weeks (range 2 weeks to 12 months) has been reported (summarized in Shotar *et al.*^8^). However, intervals up to 4 years in cases with characteristic histological findings of granulomatous lesions with foreign body material have been reported,^16^ indicating a highly variable time period between EVT and disease manifestation. Affected patients in our cohort became symptomatic with diverse symptoms: headache and focal neurological signs were prevalent in all patients, epileptic seizures and cognitive deficits in some. This is in line with other reports describing various symptoms including seizures, headache, motor deficits and visual disturbances.^8,12^ In the diagnostic workup, CSF analysis revealed a pleocytosis only in two out of five patients, consistent with the literature,^4,5,9,13,17,19^ where normal and pathological CSF findings have been reported.

The disease course of patients suffering from NICE lesions after EVT is highly variable.^2,11^ Whereas cases resolving completely without any treatment have been described,^7,11^ most patients reported were treated with GCS.^4,8,11,12,16^ In some cases, GCS treatment resulted in complete remission,^4,11,12^ but in others it did not.^2,5^ To our knowledge, there are only single reports on the use of immunosuppressants other than GCS, two reports on the use of MMF,^16,17^ one report on MTX^16^ and two reports on 4 patients treated with AZA.^13,18^

In our study, all patients received GCS, all but one immediately after confirming the diagnosis, with positive effects on MRI activity and clinical symptoms. Case 6 was treated only with GCS, all other patients received additive immunotherapies. The selection of immunosuppressants was decided based on the severity of symptoms and/or MRI findings. As additive immunosuppressants in our study, CYC, AZA, MMF, MTX, RTX and TCZ were used. As first line therapies in our cohort, less selective immunotherapies were chosen first, before monoclonal antibodies (RTX, TCZ) were given in persistent disease activity.

Regarding CYC, we found only one report^9^ on a patient treated in addition to GCS for recurrent delayed leukoencephalopathy after thrombectomy (without stenting) resulting in a stable disease course. CYC given in cases 2 and 3 (given after the first and second EVT) resulted in a reduction of NICE lesions, the patients remained symptom-free. Due to known toxicity during long-term treatment, CYC was switched to less toxic immunosuppressants after stabilization.

Treatment with AZA has been described in cerebral foreign body reaction after stenting of a carotid aneurysm,^13^ where, after recurrent symptoms following GCS discontinuation, a lower dose of oral prednisone in addition to AZA (2 mg/kilogram body weight daily) resulted in clinical stabilization. The last brain MRI was reported to show a substantial edema regression. Three patients in the study by Moreno Estébanez *et al.*^18^ were also treated with AZA (doses ranging from 75 to 250 mg daily) in addition to GCS with clinical and radiological improvement.

In our study, due to the short treatment period, the effect of AZA could not be estimated in case 3, case 2 (AZA dosage at start 125 mg daily, then up to 150 mg daily) showed clinical and repeated MRI activity under AZA prompting a treatment switch to MMF.

Because of side effects, MTX given in case 1 could only be dosed up to 7.5 mg weekly without disease control. The low dose and short treatment period of only 3 months however do not allow to estimate treatment effect reliably. As recently published, MTX up to 20 mg weekly, combined with GCS, resulted in resolution or reduction of lesions and regression of symptoms after EVT; however, after a total of 27 months of immunosuppression, new lesions were detected, so the authors considered a treatment change.^16^

For MMF uneven efficacy has been reported. In a case with polymeric cerebral granulomatous reactions receiving MMF 3 g daily after prednisone had been tapered, follow-up MRI showed ongoing nodular enhancement at year 1.^16^ Another patient treated with MMF for a second time, reinstituted due to new MRI activity, was symptom-free after more than 47 months after coiling with the last brain MRI showing no new white matter lesions.^17^

In our study, we also found variable treatment response to MMF. MMF (2 g daily) combined with GCS resulted in clinical and MRI stabilization without detectable NICE lesions in case 3 after the second and third EVT and in case 4 despite tapering prednisolone. In case 2 and 5 however, patients showed ongoing MRI activity and headache, hence treatment was switched to TCZ.

In the literature, histopathological evaluation of lesions after EVT revealed granulomatous lesions with foreign body material consistent with a type IV reaction,^16^ a non-specific lymphocytic inflammatory infiltration, reactive astrocytes, and a minimal eosinophilic leukocytoclastic vasculitis^8^ and granulomatous angiitis encasing foreign material.^5^ Based on findings of vasculitis-like changes on angiography and also in regard to MRI features in our patients resembling lesions seen in vasculitis, we used a monoclonal antibody treatment approach, RTX and TCZ, not described in the literature so far in this disorder, for unstable patients.

RTX, a monoclonal B-cell depleting antibody directed against CD20, has been shown to be effective in cases of primary angiitis of the central nervous system.^20^ In case 1 however, RTX did not result in clinical or MRI stabilization despite depleted B-cells, resulting in a treatment switch to TCZ.

TCZ, a humanized anti-interleukin-6 receptor monoclonal antibody, given subcutaneously is effective and licensed in giant cell arteritis. There are also reports showing an efficacy in anti-neutrophil cytoplasmic antibody-associated vasculitis, a systemic, pauci-immune, necrotizing small vessel vasculitis associated with circulating anti-neutrophil cytoplasmic antibodies (ANCAs).^21^ Based on these reports, we used TCZ in case 1 after RTX and case 2 after MMF was stopped due to disease activity. In case 1, no NICE lesions could be detected on follow-up MRI 4 months after treatment initiation, however, another 6 months later a single new NICE lesion without perifocal edema could be detected. Clinically, however, patient’s symptoms improved constantly, so she did not use a walking stick anymore and remained without new clinical symptoms since start of TCZ. Case 2 was clinically stable with TCZ, MRI after 3 months revealed a decreasing number of NICE lesions. Due to ongoing disease activity, TCZ was started in case 5 in September 2021. Due to the short duration of TCZ treatment in our cases (1, 4, and 10 months) firm conclusions on the efficacy of TCZ cannot be drawn.

In summary, a treatment response was seen for GCS, TCZ, and MMF (in two of four cases), whereas RTX and AZA did not result in treatment stabilization. Due to the low numbers of treated patients, firm conclusions or recommendations cannot be given. Our data however, contribute to the growing body of evidence in this rare disorder that may aid treatment decisions in similar cases. Since type and optimal duration of immunotherapy remain to be determined, individual treatment should be adapted to the clinical course, effects on MRI lesions and side effects.

In our cohort, in none of the cases immunotherapy has been withdrawn, in case 6 long-term immunotherapy was refused by the patient. Therefore, we have no data on recurrence after immunotherapy withdrawal. Bakola *et al.*^22^ reported a case with recurrence of symptomatic NICE lesions two years after slowly tapering GCS. Hence, physicians should be aware of the risk of recurrent disease activity after discontinuing immunotherapy.

The pathophysiology of NICE lesions has been subject of numerous investigations. Besides foreign body emboli, in some patients identified as hydrophilic polymer emboli, and subsequent granulomatous reactions,^2,3,5,6,8,13,14^ cerebral metallic hypersensitivity and nickel allergy^4,7,15^ have been reported. Our study does not contribute to uncover the underlying pathophysiology of NICE lesions, as no biopsy was performed in any of our case. In case 2, dermatologic testing including gene-panel analysis for systemic autoinflammatory disease and epicutaneous testing, also performed in case 6, for various metals including the metals used during EVT was not conclusive. In case 6, a lymphocyte transformation test was negative for nickel, chrome and cobalt. The results have to be interpreted with caution in case 2, since testing was done under immunotherapy (AZA 150 mg and prednisolone 5 mg daily). Of interest is case 3 presenting new NICE lesions after repeated EVT, responding to escalating immunotherapies each time, to our knowledge so far not yet reported in the literature. This may indicate that some individuals are more prone to developing NICE lesions after EVT.

The major limitations of our study are the predominantly retrospective design and the small number of cases in this single center study. The fact that we added one patient from another department may result in a selection bias. However, due to the rarity of this disorder and paucity of long-term data, we considered the inclusion of another case, we got aware of by personal communication, as informative.

In conclusion, delayed NICE lesions are a rare complication after EVT, requiring immunotherapies in all patients reported here. However, since asymptomatic patients, long term persistent enhancement and cases resolving completely without any treatment have been described in the literature, the decision to start an immunotherapy has to be made individually. Physicians should be aware of this disorder in case of new symptoms or contrast enhancing lesions after EVT.

## Supplemental Material

sj-docx-1-tan-10.1177_17562864211072372 – Supplemental material for Incidence, clinical spectrum, and immunotherapy of non-ischemic cerebral enhancing lesions after endovascular therapy Click here for additional data file. Supplemental material, sj-docx-1-tan-10.1177_17562864211072372 for Incidence, clinical spectrum, and immunotherapy of non-ischemic cerebral enhancing lesions after endovascular therapy by Antonios Bayas, Monika Christ, Ansgar Berlis, Markus Naumann, Michael Ertl, Felix Joachimski, Mona Müller, Julia Welzel, Lisa Ann Gerdes, Klaus Seelos and Christoph Maurer in Therapeutic Advances in Neurological Disorders

## References

[1] Ihn YK Shin SH Baik SK , et al Complications of endovascular treatment for intracranial aneurysms: management and prevention. Interv Neuroradiol 2018; 24: 237–245. 29466903 10.1177/1591019918758493 PMC5967192

[2] Cruz JP Marotta T O’Kelly C , et al Enhancing brain lesions after endovascular treatment of aneurysms. Am J Neuroradiol 2014; 35: 1954–1958. 24874528 10.3174/ajnr.A3976 PMC7966257

[bibr3-17562864211072372] Fealey ME Edwards WD Giannini C , et al Complications of endovascular polymers associated with vascular introducer sheaths and metallic coils in 3 patients, with literature review. Am J Surg Pathol 2008; 32: 1310–1316. 18636015 10.1097/PAS.0b013e318165582a

[bibr4-17562864211072372] Lobotesis K Mahady K Ganesalingam J , et al Coiling-associated delayed cerebral hypersensitivity: is nickel the link? Neurology 2015; 84: 97–99. 10.1212/WNL.0000000000001106 25428692

[bibr5-17562864211072372] Shapiro M Ollenschleger MD Baccin C , et al Foreign body emboli following cerebrovascular interventions: clinical, radiographic, and histopathologic features. Am J Neuroradiol 2015; 36: 2121–2126. 26294650 10.3174/ajnr.A4415 PMC7964857

[bibr6-17562864211072372] Skolarus LE Gemmete JJ Braley T , et al Abnormal white matter changes after cerebral aneurysm treatment with polyglycolic-polylactic acid coils. World Neurosurg 2010; 74: 640–644. 21492633 10.1016/j.wneu.2010.03.026

[bibr7-17562864211072372] Ulus S Yakupoglu A Kararslan E , et al Reversible intracranial parenchymal changes in MRI after MCA aneurysm treatment with stent-assisted coiling technique; possible nickel allergy. Neuroradiology 2012; 54: 897–899. 22653481 10.1007/s00234-012-1048-2

[8] Shotar E Law-Ye B Baronnet-Chauvet F , et al Non-ischemic cerebral enhancing lesions secondary to endovascular aneurysm therapy: nickel allergy or foreign body reaction? Case series and review of the literature. Neuroradiology 2016; 58: 877–885. 27216205 10.1007/s00234-016-1699-5

[9] Mellemkjaer T Chandra RV Speiser L , et al Delayed leukoencephalopathy from suspected polymer embolism after neuroendovascular procedures. Neuroradiol J 2021; 34: 373–378. 34224249 10.1177/19714009211029172 PMC8447825

[10] Shotar E Labeyrie MA Biondi A , et al Non-ischemic cerebral enhancing lesions after intracranial aneurysm endovascular repair: a retrospective French National Registry. J Neurointerv Surg. Epub ahead of print 20 September 2021. DOI: 10.1136/neurintsurg-2021-017992. 34544825

[11] Ikemura A Ishibashi T Otani K , et al Delayed leukoencephalopathy: a rare complication after coiling of cerebral aneurysms. Am J Neuroradiol 2020; 41: 286–292. 32001447 10.3174/ajnr.A6386 PMC7015205

[12] Nakagawa I Park HS Kotsugi M , et al Delayed intracranial parenchymal changes after aneurysmal coil embolization procedures for unruptured intracranial aneurysms. Oper Neurosurg 2020; 19: 76–83. 10.1093/ons/opz299 31584072

[13] Lorentzen AO Nome T Bakke SJ , et al Cerebral foreign body reaction after carotid aneurysm stenting. Interv Neuroradiol 2016; 22: 53–57. 26510943 10.1177/1591019915609171 PMC4757375

[14] Mehta RI Mehta RI Solis OE , et al Hydrophilic polymer emboli: an under-recognized iatrogenic cause of ischemia and infarct. Mod Pathol 2010; 23: 921–930. 20305613 10.1038/modpathol.2010.74

[15] Tsang ACO Nicholson P Pereira VM . Nickel-related adverse reactions in the treatment of cerebral aneurysms: a literature review. World Neurosurg 2018; 115: 147–153. 29684517 10.1016/j.wneu.2018.04.073

[16] Boyle T Fernando SL Steinfort B , et al Medical treatment of polymeric cerebral granulomatous reactions following endovascular procedures. J Neurointerv Surg 2021; 13: 1032–1036. 33722971 10.1136/neurintsurg-2020-016806

[17] Grewal SS Lopez Del Valle EM Gupta V , et al Neurological changes with abnormal brain reactivity following coiling of cerebral aneurysm. possible reactivity to endovascular devices and material? J Vasc Interv Neurol 2015; 8: 28–36. PMC4535604 26301029

[18] Moreno Estébanez A Luna Rodríguez A Pérez Concha T , et al Symptomatic intracranial embolic foreign-body reactions after endovascular neurointerventional procedures: a retrospective study in a tertiary hospital. Clin Neurol Neurosurg 2021; 200: 106323. 33158631 10.1016/j.clineuro.2020.106323

[19] Deguchi K Kawahara Y Deguchi S , et al A patient develops transient unique cerebral and cerebellar lesions after unruptured aneurysm coiling. BMC Neurol 2015; 15: 49. 25884179 10.1186/s12883-015-0303-7 PMC4387739

[20] De Boysson H Arquizan C Guillevin L , et al Rituximab for primary angiitis of the central nervous system: report of 2 patients from the French COVAC cohort and review of the literature. J Rheumatol 2013; 40: 2102–2103. 24293623 10.3899/jrheum.130529

[21] Sakai R Kondo T Kurasawa T , et al Current clinical evidence of tocilizumab for the treatment of ANCA-associated vasculitis: a prospective case series for microscopic polyangiitis in a combination with corticosteroids and literature review. Clin Rheumatol 2017; 36: 2383–2392. 28733791 10.1007/s10067-017-3752-0

[22] Bakola E Katsanos AH Palaiodimou L , et al Delayed recurrent enhancing white matter lesions complicating coiling of intracranial aneurysm. Eur J Neurol 2021; 28: 2388–2391. 33780579 10.1111/ene.14844

