# Supplementary material for: Incidence, clinical spectrum, and immunotherapy of non-ischemic cerebral enhancing lesions after endovascular therapy
Source: Ther Adv Neurol Disord. 2022 Jan 31;15:17562864211072372. doi: 10.1177/17562864211072372 (PMC8808010; doi:10.1177/17562864211072372)
Supplement: sj-docx-1-tan-10.1177_17562864211072372 – Supplemental material for Incidence, clinical spectrum, and immunotherapy of non-ischemic cerebral enhancing lesions after endovascular therapy [file sj-docx-1-tan-10.1177_17562864211072372.docx]

**Supplementary Appendix 1**

Case descriptions

**Case 1:**

A 35-year-old female, with no relevant medical history except migraine with aura, underwent a stent-assisted coil embolization (SACE) of two incidental aneurysms of the left supraophthalmic internal carotid artery (ICA) and the right ICA in July 2018. Approximately 41 days after endovascular therapy (EVT), the patient presented with progressive headache, concentration difficulties, right-sided hemiparesis, hypesthesia of the right hand and fingertips on the left, dysarthria, and aphasia. Cranial magnetic resonance imaging (MRI) showed multiple non-ischemic cerebral enhancing (NICE) lesions with extensive perifocal edema, in the left middle cerebral artery (MCA) territory and the right anterior cerebral artery (ACA) territory, and less prominent in other parts of the anterior circulation both sides and in the territory of the posterior cerebral artery (PCA) both sides (intermediate-type PCA on both sides). Magnetic resonance angiography (MRA) revealed no signs of vasculitis. Since all clinical and laboratory workup was negative for infection, the patient was treated with high dose methylprednisolone (MP), followed by oral prednisolone tapering to 20 mg/day resulting in a marked clinical improvement with persistent mild concentration disturbance and reduced fine motor skills right hand. Methotrexate (MTX) was started, due to side effects the maximum dose was 7.5 mg/week subcutaneously (SC). MRI 8 days after starting immunotherapy, showed a reduced NICE lesion count. However, approximately 4 months after disease onset, the patient deteriorated clinically (increasing headache, sensory symptoms right facial, right hand as well as fingertips on the left side), MRI showed new NICE lesions left frontal, left hippocampal as well as right occipital. Cerebrospinal fluid (CSF) analysis was normal, serologic vasculitis parameter negative. After high dose MP, MTX was switched to rituximab (RTX) (1 g 2 weeks apart, followed by 1 g every 6 months intravenously (IV)), resulting in clinical and MRI improvement. Despite depleted B-cells, repeated disease activity, clinical and on MRI, occurred 9, 11 and 21 months after starting RTX. Treatment was switched to tocilizumab (TCZ) (8 mg/kg body weight IV every 4 weeks) in December 2020. Patient has been stable, with no NICE lesions in the MRI in March 2021. Prednisolone could be tapered to 1,5 mg/day. Eight months after starting TCZ, the dose had to be reduced temporarily to 7 mg/kg body weight once, because of an asymptomatic liver enzymes increase (ALT (alanine aminotransferase) 101 U/l (reference < 35 U/l), AST (aspartate aminotransferase) 48 U/l (reference < 35 U/l), Gamma-Glutamyl Transferase (GGT) 46 U/l (reference < 42 U/l). At the last follow up 10 months after starting TCZ, the patient was still clinically stable; MRI was unremarkable except for one new singular punctate contrast enhancing lesion in the left parietal lobe without edema. Liver enzymes had improved in the meantime and TCZ was continued with 8 mg/kg body weight IV every 4 weeks.

**Case 2:**

A 52-year-old woman was admitted for EVT of 3 incidental aneurysms at the bifurcation of the right and left medial bifurcation of the MCA, and the ramus communicans anterior (AcomA). The patient had a medical history of nicotine abuse, allergic asthma (allergies against aspirin, cow's milk, soy, nuts, pome fruits, animal dander, pollen), livedo reticularis since childhood and a surgically treated ovarian cancer years ago. The AcomA aneurysm was treated by SACE and the two MCA aneurysms with a Woven EndoBridge (WEB) device. Sixteen days after EVT, the patient developed progressive headache and a left-sided arm paresis. On admission, 17 days after symptom onset, MRI showed multiple, predominantly cortical enhancing lesions with perifocal edema. CSF analysis revealed a lymphocytic pleocytosis (37 cells/µl; glucose normal, protein 0.66 g/l, normal range 0.15–0.45 g/l), negative oligoclonal IgG bands). Blood and CSF tests for bacterial or viral infections were negative, as well as the laboratory parameters for rheumatological diseases and a panel of anti-neuronal autoantibodies in serum and CSF. Neurocognitive testing and electroencephalogram (EEG) were unremarkable. High-dose MP was administered, followed by oral tapering. Cyclophosphamide (CYC) was initiated (350 mg/m^2^ body surface/day IV for 3 consecutive days, then 600 mg/m^2^ body surface IV every 4 weeks). Few days after starting immunotherapy, headache and arm paresis resolved completely, prednisolone was gradually tapered to 5 mg/day. Follow-up MRIs in the following 4 months showed a continuous reduction of NICE lesions and the patient remained symptom-free. CYC was switched to AZA after 6 months (up to 125 mg daily). After initially stable disease course, follow-up MRIs 6 and 10 months after starting AZA showed increasing NICE lesion counts, after AZA dose increase to 150 mg daily patient was transiently stable. Sixteen months after AZA treatment initiation, CSF cell count was normal, protein mildly elevated (0.66 g/l). Dermatologic testing around 1 month later, including gene-penal analysis for systemic autoinflammatory disease and epicutaneous testing for various metals and the stents, WEBs, microcatheters, and coils used during EVT were performed and were unremarkable except for an amalgam allergy. After 22 months, AZA was switched to mycophenolate mofetil (MMF) (2 g/day), due to persisting disease activity on MRI. However, NICE lesion count increased and headache reappeared after 9 months, treatment therefore was changed to TCZ (8 mg/kg IV every 4 weeks). After 4 infusions, patient was clinical stable, MRI showed marked improvement with regressive edema and regressive number of enhancing lesions. Prednisolone was tapered to 5 mg daily.

**Case 3:**

A 51-year-old female suffered from stroke in the territory of the right middle cerebral artery (MCA) with dysarthria, visual disturbance, and headache in September 2009, most likely due to an arterio-arterial embolism from a newly diagnosed, partially thrombosed MCA aneurysm. CSF analysis was normal except a slightly elevated protein (0.63 g/l). The symptomatic aneurysm, as well as a small asymptomatic right MCA aneurysm at the proximal M1 segment and a right pericallosal aneurysm were treated by SACE, three additional incidentally detected aneurysms remained untreated (2 left MCA, 1 right ACA). Twentythree days after the intervention, the patient developed progressive headache and left-sided hemiparesis, homonymous hemianopsia and neglect. MRI showed disseminated NICE lesions in the MCA territory on the right, but also on the left with surrounding edema. CSF analysis now revealed a lymphomonocytic pleocytosis (56 leukocytes/µl) with elevated protein (1,1 g/l), oligoclonal IgG bands were negative. Additional laboratory assessment was not indicative for systemic vasculitis. Testing for bacterial and viral infections in serum and CSF was negative. A follow-up MRI, 2 weeks after symptom onset, showed increasing NICE lesion count and digital subtraction angiography (DSA) revealed new and marked intracranial caliber irregularities with stenosis of peripheral branches, particularly of the right MCA, so the patient was treated with high-dose MP followed by oral prednisolone tapering for suspected vasculitis. Symptoms improved with persistent mild distal paresis of the left arm as well as a visual and sensory neglect to the left, CSF pleocytosis decreased to 33 leukocytes/µl and MRI showed decreasing numbers of NICE lesions about one month later. Prednisolone was gradually tapered to 4 mg/day and the patient was treated with CYC (350 mg/m^2^ body surface /day IV for 3 consecutive days, then 600 mg/m^2^ body surface IV every 4 weeks) for one year, before therapy was switched to AZA (200 mg daily) free of disease activity. In December 2010, 6 weeks after cessation of CYC, the patient underwent another SACE of previously untreated aneurysms of the left distal MCA and left proximal MCA without complications, coiling of a left pericallosal artery (PcaA) aneurysm and a recurrence of the right MCA aneurysm. Ten days thereafter, left-sided hemiparesis worsened, MRI showed increasing NICE lesions, especially in the right MCA territory. AZA was discontinued, the patient was treated with high-dose MP and CYC was continued (600 mg/m^2^ body surface IV every 4 weeks), together with prednisolone. After another 7 months of CYC and stable disease, treatment was switched to MMF (2 g/day). Over the following 5 years, there was, except for a transient episode of headache, no disease activity, so MMF was gradually tapered and discontinued 2016. A monotherapy with prednisolone (5 mg/ day gradually tapered to 1 mg/day) was continued, leading to a stable course for the following 3 years. In December 2019, an increase in size of the right-sided MCA aneurysm necessitated repeat EVT. The aneurysm was treated by a flow diverter, peri-interventionally the oral steroid dose was increased. Fourteen days later the patient presented with progressive headache, MRI revealed new NICE lesions. Therapy with MMF was resumed (gradually escalating to 2 g/day), prednisolone was increased to 20 mg/day. At the last follow-up in October 2020, 11 months after the last EVT, the patient was free from headache, in MRI NICE lesions had resolved, prednisolone had been reduced to 5 mg daily in between.

**Case 4:**

A 73-year-old otherwise healthy female suffered from recurrent attacks of dizziness and double vision. MRI revealed a large, partially thrombosed basilar aneurysm with space-occupying effect and mild compression of the left pons. The aneurysm was treated by SACE, episodes of double vision and dizziness resolved. Four days after EVT, the patient developed progressive headache, confusion, transient visual disturbances, and dizziness. MRI, 16 days after EVT, displayed multiple nodular enhancing lesions bilateral in the brainstem, cerebellum, temporooccipital and parietal without diffusion restriction. Oral prednisolone was started (60 mg/day), followed by MMF (gradually increased to 2 g/day) 3 months later. Repeated follow-up MRIs demonstrated decreasing numbers of NICE lesions, prednisolone was tapered to 5 mg/day. Twentyone months after starting immunotherapy, the patient had no residual symptoms and MRI was free of NICE lesions.

**Case 5:**

A 43-year-old female with a history of migraine without aura was admitted for EVT of two asymptomatic aneurysms of the right supraophthalmic ICA and left paraophthalmic ICA. The right ICA aneurysm was treated by SACE and the left ICA aneurysm was occluded by a flow diverter. Two days after the procedure, the patient developed previously unknown progressive occipital headache and about one month after the EVT non-convulsive focal seizures left side. During the following two months, headache worsened, and the patient developed a paresis of the left hand. At the pre-planned follow-up DSA three months after EVT, both aneurysms were successfully treated, but subtle irregularities of the peripheral arteries in the treated vascular territory were observed. MRI showed multiple NICE lesions in the entire right hemisphere, marked parietooccipital (fetal posterior cerebral artery (PCA) on the right side), and in the territory of the left ACA and MCA. CSF analysis was normal, additional laboratory tests were normal except for an earlier hepatitis B infection (hepatitis B virus (HBV)- DNA negative). Antiepileptic treatment with levetiracetam and high-dose MP, followed by oral prednisolone tapering was initiated. Headache and the paresis of the left hand improved, but the frequency of the focal seizures increased, so levetiracetam was gradually increased and lamotrigine added, resulting in a reduction in seizure frequency. MMF was initiated (gradually dosed up to 2 g/day) and prednisolone was gradually tapered to 20 mg/day. The first follow-up MRI 6 weeks after starting immunotherapy showed a reduced number of NICE lesions. But despite antiepileptic treatment, the focal seizure frequency increased and about 2 months after diagnosis the patient suffered from a secondary generalised epileptic seizure and presented with progressive headache and a new hemianopsia to the left. MRI revealed multiple new NICE lesions with progressive edema, especially right parietooccipital. Another course of high-dose MP was given, followed by prednisolone 1 mg/kg/day orally, which was gradually tapered to 20 mg/day. Additionally, lamotrigine was dosed up as planned. One month later, headache increased again transiently, and the patient suffered from two focal epileptic seizures. MRI showed only a slight decrease in the NICE lesion count. Lamotrigine was increased. However, after 2 months, headache increased again, and the patient suffered from fatigue and repeated daily focal seizures. MRI revealed a slight decrease in the NICE lesion count but few new NICE lesions right hemisphere. Treatment with MMF was switched to TCZ (8 mg/kg IV every 4 weeks), prednisolone was continued with 20 mg/day and lamotrigine dosage was further increased.

**Case 6:**

The clinical course of the 54-year-old woman has partially been presented previously.^1^ Her medical history did not include any relevant diseases except hyperlipidemia and nicotine abuse. MRA was performed due to suspected right anterior ischemic optic neuropathy and revealed an incidental right supraophthalmic ICA aneurysm which was treated by flow diverter. Post-interventional MRI the day after the procedure showed few, small diffusion-restricted lesions in the right frontal brain in the vascular territory of the treated aneurysm and was considered as a periprocedural finding. Five days after EVT and again one month later, the patient presented with new symptoms. She suffered from right temporal and retrobulbar headaches and intermittent left brachiofacial paraesthesias, which had increased in frequency and severity over time. MRI showed several subcortical enhancing nodular lesions in the right frontal and parietal lobes with perilesional edema, the diffusion-restricted lesions were no longer detectable. Differential workup ruled out infection or any other underlying autoimmune condition (pathological findings of slightly elevated antinuclear antibodies (ANA) at 1:400 (granular pattern), IgM anti-cardiolipin (18,5 MPL-U/ml, reference <12 MPL-U/ml) and anti-beta2-glycoprotein antibodies (371 U/ml, reference <20 U/ml) without any clinical correlation) and a foreign body reaction was suspected. EEG was normal, but due to suspected focal epileptic seizures anticonvulsive treatment with levetiracetam was initiated and achieved complete clinical remission. During follow-up, MRI 4 months after EVT showed almost complete regression of the NICE lesions, however at month 13 and 21 a significant increase in right hemispheric NICE lesions was detectable. Further workup, including brain scintigraphy before and after acetazolamide administration and repeated CSF analysis, was unremarkable and the patient remained asymptomatic. Twentythree months after intervention, the patient developed new headaches, weakness in both legs, dizziness, unsteadiness of gait, hypaesthesia and coordination difficulties of the left hand as well as a pulse-synchronous tinnitus and depressive mood. MRI again demonstrated new NICE lesions right frontal and parietal. In O-(2- [18F]fluoroethyl)-L-tyrosine (18F-FET) positron emission tomography (PET) lesions were partly positive for 18F-FET uptake, consistent with an inflammatory origin. Furthermore, DSA revealed multiple stenoses, typical for vasculitis, of the distal pial arteries and cortical branches of the right ACA and MCA, so high-dose MP was administered, followed by oral prednisolone tapering. One month after starting immunotherapy, headache had improved, the patient showed no focal deficit except for mild cognitive impairment in neurocognitive testing. While MRI showed a partial reduction of NICE lesions, the overall lesion load continued to increase. Dermatologic epicutaneous testing for various metals, including the metals used during the EVT, was unremarkable. In the following 6 years, she presented several times for worsening headaches, once with accompanying sensorimotor disturbance of the left side for 30 minutes and was admitted due to focal status epilepticus of the left hand. MRI repeatedly demonstrated new NICE lesions, always in the right hemisphere. Prednisolone therapy though recommended, was taken only inconsequently, any further immunotherapy or antiepileptic treatment was refused. Last MRI, 106 months after EVT, still demonstrated, albeit declining, NICE lesions right frontal and parietal. At the last follow-up 123 months after EVT, the patient was free from headache.

References

1. Cruz JP, Marotta T, O'Kelly C, et al. Enhancing brain lesions after endovascular treatment of aneurysms. AJNR Am J Neuroradiol 2014; 35: 1954–1958.
